# A U-shaped association between sleep duration and depression in adult prescription opioid users

**DOI:** 10.3389/fpsyt.2026.1755263

**Published:** 2026-03-12

**Authors:** Ying Li, Yuting Zhong, Wenting Guan, Luwen Liang, Yunya Zhu

**Affiliations:** 1 Department of Anesthesiology, The First Affiliated Hospital of Gannan Medical University, Ganzhou, China; 2 General Practice School of Gannan Medical University, Ganzhou, China; 3 Department of General Practice, the First Affiliated Hospital of Gannan Medical University, Ganzhou, China

**Keywords:** depression, NHANES, prescription opioid, sleep duration, statistical analysis

## Abstract

**Background:**

The objective of this study was to investigate the correlation between sleep duration and depression in adult prescription opioid users.

**Methods:**

Data on adults who had recently used prescription opioids were collected from the National Health and Nutrition Examination Survey (NHANES) in the United States, covering the period from 2007 to 2018. Depressive symptoms were assessed using the PHQ-9 scale. A multivariate logistic regression model was employed to examine the independent association between sleep duration and the occurrence of depression. To explore the potential non-linear relationship between sleep duration and depression, a restrictive cubic spline analysis was applied. Finally, propensity score matching (PSM) was utilized to validate our findings based on external datasets.

**Results:**

This study was conducted on a population of 1319 adult users of prescription opioids, with a weighted total of 7319533.25 individuals. 23.2% of the subjects satisfied the diagnostic criteria for depression. After adjusting for all confounding variables, the prevalence of depression was 60% greater among individuals with insufficient sleep duration [OR = 1.60, 95% CI = 1.11-2.29, P = 0.012] in comparison to those with a normal sleep length. The subgroup analyses revealed that the connection between short sleep duration and depression was considerably influenced by both gender and lower socioeconomic status, as indicated by a statistically significant interaction effect. We identified a U-shaped correlation between sleep duration and depression scores using a two-segment linear regression model, with a turning point at 6.74 hours. The PSM analysis further revealed that the less than 6.74 hours of sleep time among opioid users was closely related to depression.

**Conclusions:**

Only Insufficient sleep is associated with an increased risk of depression, and sleep duration showed a nonlinear dose response relationship in adult prescription opioid users.

## Background

Depression is a significant and growing contributor to the global burden of disease, and it is linked to premature mortality, functional disability, and a reduced quality of life (1, 2). In 2019, depression affected approximately 280 million individuals, which is equivalent to approximately 5% of the global population, according to the World Health Organization’s report (3). Concurrently, it imposed an immense financial burden on the public healthcare system (4).

Studies suggest that there may be a reciprocal relationship between the likelihood of depression and the consumption of prescription opioids (5–7). Prescription analgesics, such as buprenorphine and tramadol, are commonly used to manage depressive symptoms (8, 9). Nevertheless, they may contribute to feelings of sadness or raise the risk of depression relapse among individuals using prescription opioids. Krause et al. found that both opioid misuse and opioid use were linked to more severe depressive symptoms and a higher prevalence of probable major depression (10). Scherrer et al. found that stable, gradual, and accelerated increases in prescription opioid dosages were linked to the development of new-onset depression (11). Therefore, it is crucial to identify key factors that could help better understand and mitigate the risk of depression linked to prescription opioid use. Several studies have highlighted the significant impact of sleep duration on overall mental health (12, 13). Inadequate sleep patterns are well-documented to have a range of harmful effects.

Epidemiological and meta-analytic studies provide strong evidence for a causal link between both short and long sleep durations and various negative health outcomes (14–16). A considerable body of research has investigated the impact of sleep duration on the development of depression. Multiple studies have indicated that both short and long sleep durations are strongly associated with an increased risk of depression in adults (14, 17). Further studies support the idea that both insufficient and excessive sleep increase the risk of developing depression (18, 19). However, the relationship between sleep duration and depression in adult prescription opioid user is unclear. The National Health and Nutrition Examination Survey (NHANES) is a broad cross-sectional study that collects data on the health and nutrition of the United States household population (https://www.cdc.gov/nchs/nhanes/index.htm). Offering a wealth of information on population demographics, socioeconomic status, diet, health indicators, physiological assessments, laboratory tests, and more, the NHANES database provides comprehensive coverage and a diverse range of metrics from across the United States (20). This study identified the relationship between sleep duration and both depression scores and the prevalence of depression in adults using prescription opioids, based on data from the NHANES large-scale cross-sectional survey. This relationship may serve as a valuable predictive indicator for depression.

## Methods

### Research population

**Figure 1:**
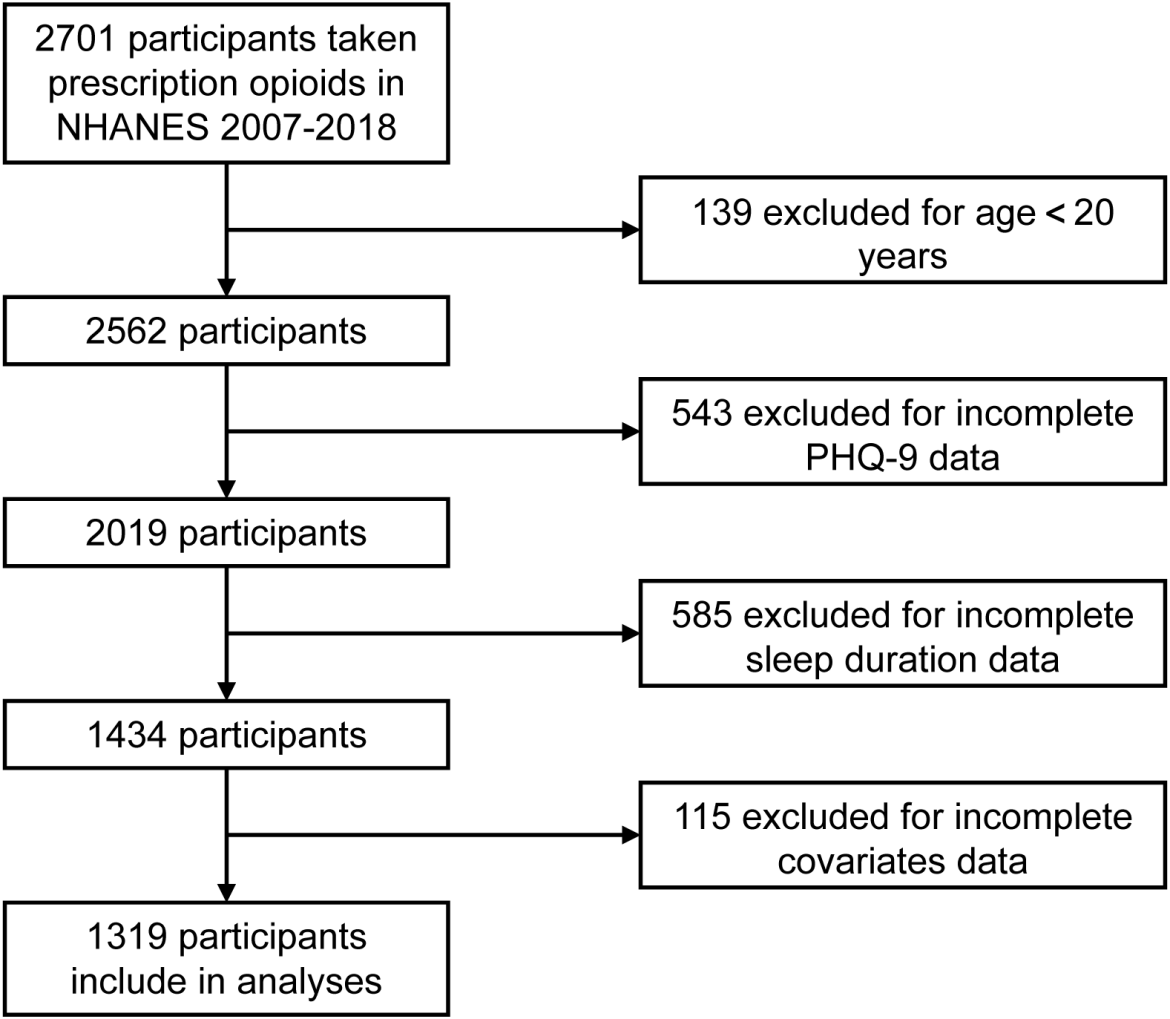
Screening of admissions for inclusion.

The data used in this study were sourced from the official NHANES website (https://www.cdc.gov/nchs/nhanes/index.htm). NHANES, a comprehensive survey conducted by the Centers for Disease Control and Prevention (CDC) and the National Center for Health Statistics (NCHS), provides nationally representative statistics on the civilian population of the United States. A dataset for this study was created by extracting responses from the publicly available NHANES data files spanning from 2007 to 2018 (**Figure 1**). Every two years, NHANES conducted cross-sectional surveys to collect a sample representing the U.S. population. Specific sampling strategies were employed for this purpose. The study included all adults aged 20 years or older who provided detailed information on their opioid prescription history via a medication questionnaire. Additionally, all participants completed the Patient Health Questionnaire (PHQ-9). A total of 1,319 participants were included after excluding those with incomplete covariate data. Finally, A total of 229 adult patients using opioid medications in our hospital were ultimately selected as research subjects. Based on the results of the RCS analysis, their sleep duration was categorized into two groups. Subsequently, PSM analysis was conducted to further examine the relationship between sleep duration and the occurrence of depression. The data collection procedures for NHANES were approved by the NCHS Ethics Review Board, and all participants provided informed consent before their interviews and tests.

### Prescription opioid

The opioid medication history prior to the interview was gathered using the Prescription Medications Questionnaire. To identify the different types of prescription opioids, the Multium drug classification codes 60 (anesthetic/analgesics) and 191 (anesthetics and analgesics) were utilized (21, 22). **Supplementary Table 1** provides a detailed list of generic drug names and their corresponding NHANES codes for each drug category. Participants are classified as short-term users (< 90 days) or long-term users (≥ 90 days) according to the duration of consecutive days they have used the medication prior to completing the questionnaire (22, 23).

### Exposure variable

Participants self-reported the amount of sleep they typically received on workdays or weekdays. From 2005 to 2014, daily sleep duration for NHANES participants was assessed with the question, “How much sleep do you get (hours)?” For the 2015–2018 cycle, sleep duration was defined using the question, “How much sleep (do you/does SP) usually get at night on weekdays or workdays?” The reported sleep durations were categorized into three groups: long (greater than 9 hours per night), normal (7 to 9 hours per night), and short (less than 7 hours per night) (24).

### Outcome variable

Depression-related symptoms over the previous two weeks were assessed using the PHQ-9 during face-to-face interviews conducted at the Mobile Examination Center (MEC). Participants’ symptoms were rated on the PHQ-9, with scores ranging from 0 (not at all) to 3 (nearly every day). The total score was derived by summing the individual item scores, yielding a possible range from 0 to 27. In accordance with the DSM-IV criteria for depression, a PHQ-9 score of 10 or higher was used to define depression in this study, as specified on the official NHANES website (25, 26).

### Other covariates

The initial screening questionnaire was used by interviewers to obtain the participant’s birthdate and other demographic data through self-report. The continuous variable was age in years. Gender was indicated as either male or female. Race was categorized as Mexican American, other Hispanic, non-Hispanic white, non-Hispanic black, or other. The formula for calculating body mass index (BMI) is as follows: BMI = weight (kg)/height^2^ (m^2^). Following this, it was divided into four categories: underweight (< 18.5 kg/m^2^), normal weight (18.5–25 kg/m^2^), overweight (25–30 kg/m^2^), and obese (≥ 30 kg/m^2^) (27). Education attainment was evaluated through the initial screening questionnaire and classified as Following: Less than high school, High school, and College or higher. Marital status was organized into three categories: Never married, Widowed/Divorced/Separated, and Married/Cohabiting. The poverty income ratio (PIR) is used to categorize household income into three groups: PIR <1.5, 1.5≤ PIR <3.5, and PIR ≥3.5. The study sample was divided into four distinct categories based on their alcohol consumption patterns: non-drinker, 1 to <5 drinks/month, 5 to <10 drinks/month, and 10+ drinks/month. Smoking status (never, current, and former). Smoking status was determined by the quantity and duration of cigarettes consumed over the course of one’s lifespan. There are three distinct categories of individuals who smoke: “current,” “former,” and “never.” (Never, smoked fewer than 100 cigarettes; former, smoked more than 100 cigarettes in their lifetime and do not smoke currently; current, smoked more than 100 cigarettes in their lifetime and smoke on some days or every day). Medical professionals conducted diagnoses of various diseases, such as CVD and cancer or malignancy, by inquiring participants with the question, “Have you ever been told by a doctor or health professional that you have :?”. Diabetes was defined as a hemoglobin A1c level of 6.5% or higher or a fasting plasma glucose level of 126 mg/dl or higher, or the current administration of insulin or diabetes medications (28). Self-reported hypertension history, the use of antihypertensive medication, a systolic blood pressure (SBP) of 140mmHg or higher, or a diastolic blood pressure (DBP) of 90mmHg or higher, is the diagnostic criteria. Dyslipidemia was defined as a triglyceride level of 150 mg/dl or higher or a high-density lipoprotein cholesterol level of 40 mg/dl or lower, as determined by the National Cholesterol Education Program Adult Treatment Panel III, or by a doctor’s diagnosis or the current use of cholesterol-lowering medications (29).

### Statistical analysis

A sophisticated, multistage probability sampling approach is used to select a sample that accurately represents the civilian non-institutionalized population of the United States, ensuring the representativeness of the NHANES data (30). This study combined data from multiple survey cycles following the analytic protocols set forth by the Centers for Disease Control and Prevention, utilizing the full 2-year MEC exam weight provided by the NHANES database. The weighted sample was employed to estimate the proportions of each variable in both the depression and non-depression groups. To compare the baseline characteristics between the two groups, the chi-squared test with Rao and Scott’s second-order correction was applied.

Four weighted logistic regression models were constructed, each adjusted for different sets of variables. The odds ratio (OR) and 95% confidence intervals (CI) were calculated to assess the association between various sleep duration categories and the likelihood of depression. Model 1 was an unadjusted analysis. Model 2 included adjustments for age and gender. Model 3 accounted for age, gender, race, BMI, education level, marital status, PIR, alcohol consumption, smoking habits, physical activity, hypertension, dyslipidemia, diabetes, CVD, and history of cancer or malignancy. Building on Model 3, Model 4 included additional adjustments for opioid use, specifically for both the use of multiple opioids and long-term opioid use. In addition, participants were stratified into subgroups based on sociodemographic factors and duration of opioid use, with each subgroup analyzed using Model 4. Finally, a restricted cubic spline analysis was applied to Model 4 to examine the potential non-linear relationship between sleep duration and depression. All statistical analyses were performed using R software (version 4.3.0). A *P* < 0.05 is considered statistically significant.

## Results

### Description of the study population

In this study, 1319 participants had a recent history of taking prescription opioid, representing 7319533.25 people in the United States after weighting, and all had complete survey data. The weighted median age of participants was 51 years. Among these included participants, the male percentage was 55.8%, the female percentage was 44.2%. In the study population, 44.3% of the participants had 7 to 9 h of sleep, 48.0% slept fewer than 7h, and 7.7% slept > 9h. 23.2% of participants met the diagnostic criteria for depression by PHQ-9. Higher PHQ-9 score subjects were more likely to be male, lower education, widowed/divorced/separated, have a lower yearly household income, current smoker, lower daily physical activity, hypertension, diabetes, long-term opioid use and short sleep duration (**Table 1**).

**Table 1:** Weighted characteristics of NHANES participants, 2007–2018.

Covariates	All participant	No depression	Depression	*P*
Unweighted number	1319	982	337	
Weighted number	7319533.25	5618217.49	1701315.75	
Age, years	51.00 (41.00, 62.00)	51.00 (39.00, 63.00)	52.00 (45.00,59.77)	0.518
Gender				0.001
Male	4080786.1 (55.8%)	2941152.3 (52.4%)	1139633.9 (67.0%)	
Female	3238747.1 (44.2%)	2677065.2 (47.6%)	561681.9 (33.0%)	
Race				0.149
Mexican American	414061.1 (5.7%)	306131.5 (5.4%)	107929.6 (6.3%)	
Non-Hispanic Black	821002.2 (11.2%)	584928.7 (10.4%)	236073.5 (13.9%)	
Non-Hispanic White	5532412.4 (75.6%)	4333975.9 (77.1%)	1198436.5 (70.4%)	
Other Hispanic	229706.3 (3.1%)	165951.5 (3.0%)	63754.8 (3.7%)	
Other/multiracial	322351.3 (4.4%)	227229.9 (4.0%)	95121.4 (5.6%)	
BMI				0.068
Underweight	110033.2 (1.5%)	81975.3 (1.5%)	28057.9 (1.6%)	
Normal weight	1563734.6 (21.4%)	1261317.9 (22.5%)	302416.7 (17.8%)	
Overweight	2222887.7 (30.4%)	1770916.6 (31.5%)	451971.1 (26.6%)	
Obesity	3422877.8 (46.8%)	2504007.7 (44.6%)	918870.1 (54.0%)	
Education level				0.001
Less than high school	3422877.8 (46.8%)	2504007.7 (44.6%)	918870.1 (54.0%)	
High school	1857140.6 (25.4%)	1324178.4 (23.6%)	532962.2 (31.3%)	
College or above	3771645.6 (51.5%)	3081945.5 (54.9%)	689700.1 (40.5%)	
Marital status				< 0.001
Married/Cohabiting	4304978.8 (58.8%)	3499860.0 (62.3%)	805118.8 (47.3%)	
Widowed/Divorced/Separated	2096893.3 (28.6%)	1411364.4 (25.1%)	685528.9 (40.3%)	
Never married	917661.1 (12.5%)	706993.1 (12.6%)	210668.1 (12.4%)	
PIR				< 0.001
PIR≤ 1.3	2353822.9 (32.2%)	1578614.5 (28.1%)	775208.4 (45.6%)	
PIR (1.3 -3.5]	2797177.9 (38.2%)	2089507.3 (37.2%)	707670.5 (41.6%)	
PIR> 1.3	2168532.4 (29.6%)	1950095.7 (34.7%)	218436.8 (12.8%)	
Alcohol intake				0.440
Non-drinker	1429388.6 (19.5%)	1078180.2 (19.2%)	351208.4 (20.6%)	
1–5 drinks/month	4430642.2 (60.5%)	3352872.3 (59.7%)	1077769.8 (63.3%)	
5–10 drinks/month	417887.8 (5.7%)	327884.8 (5.8%)	90003.0 (5.3%)	
10+ drinks/month	1041614.7 (14.2%)	859280.2 (15.3%)	182334.5 (10.7%)	
Smoking status				< 0.001
Never	4678837.3 (63.9%)	3835902.9 (68.3%)	842934.5 (49.5%)	
Current smoker	2234556.9 (30.5%)	1502871.8 (26.7%)	731685.0 (43.0%)	
Former smoker	406139.0 (5.5%)	279442.8 (5.0%)	126696.2 (7.4%)	
Daily physical activity	2379592.7 (32.5%)	1930665.1 (34.4%)	448927.7 (26.4%)	0.026
Hypertension	3586838.0 (49.0%)	2523366.8 (44.9%)	1063471.2 (62.5%)	< 0.001
Dyslipidemia	4460279.0 (60.9%)	3368346.9 (60.0%)	1091932.1 (64.2%)	0.223
Diabetes	1560000.2 (21.3%)	1085614.8 (19.3%)	474385.4 (27.9%)	0.010
CVD	395085.7 (5.4%)	299961.8 (5.3%)	95123.9 (5.6%)	0.889
Cancer or malignancy	1244210.6 (17.0%)	948192.9 (16.9%)	296017.7 (17.4%)	0.865
Multiple opioid use				0.109
One type	6399296.1 (87.4%)	4963866.1 (88.4%)	1435430.0 (84.4%)	
Two types or above	920237.1 (12.6%)	654351.4 (11.6%)	265885.7 (15.6%)	
Long-term opioid use				< 0.001
< 90 d	2073953.6 (28.3%)	1817814.7 (32.4%)	256138.9 (15.1%)	
≥ 90 d	5245579.7 (71.7%)	3800402.8 (67.6%)	1445176.9 (84.9%)	
Sleep duration (h)	7.00 (6.00, 8.00)	7.00 (6.00, 8.00)	6.00 (4.00, 8.00)	0.003
Hours of sleep				0.006
<7 h	3512561.9 (48.0%)	2546407.0 (45.3%)	966154.9 (56.8%)	
7–9 h	3243439.1 (44.3%)	2665630.3 (47.4%)	577808.8 (34.0%)	
≥9 h	563532.3 (7.7%)	406180.2 (7.2%)	157352.0 (9.2%)	

### Associations between sleep duration and depression

**Table 2** presents the association between sleep duration and depression. odds ratios (ORs) (95% CI) of depression are presented for short and long sleep duration compared to the mid-range sleep duration category. In the non-adjusted model, participants who had short sleep duration had a 75% increased risk in the odds of the development of depression (OR = 1.75 [95% Cl 1.19-2.58]). In the age and gender model, participants who had short sleep duration had a 81% increased risk in the odds of the development of depression (OR = 1.81 [95% Cl 1.22-2.68]). After adjustment for confounding factors in **Table 2**, the ORs were 1.58 (1.11-2.25) and 1.60 (1.11-2.29) respectively (*P* < 0.05).

**Table 2:** Relationship between different sleep duration group and depression in the unadjusted and adjusted logistic regression models.

Groups	β	SE	Wald	OR (95%CI)	*P*	*P* for trend
Model 1^a^						
Sleep duration	-0.178	0.071	6.335	0.84 (0.73-0.96)	0.012	
Hours of sleep						0.006
< 7 h	0.560	0.194	8.345	1.75 (1.19-2.58)	< 0.001	
7–9 h				Ref		
≥9 h	0.581	0.260	4.981	1.79 (1.06-3.01)	0.029	
Model 2^b^						
Sleep duration	-0.188	0.073	6.567	0.83 (0.72-0.96)	0.010	
Hours of sleep						0.007
< 7 h	0.591	0.197	9.026	1.81 (1.22-2.68)	0.003	
7–9 h				Ref		
≥9 h	0.509	0.274	3.455	1.66 (0.96-1.01)	0.068	
Model 3^c^						
Sleep duration	-0.152	0.067	5.061	1.11 (0.98-1.27)	0.113	0.031
Hours of sleep						0.030
< 7 h	0.457	0.174	6.937	1.58 (1.11-2.25)	0.013	
7–9 h				Ref		
≥9 h	0.251	0.303	0.688	1.29 (0.69-2.38)	0.413	
Model 4^d^						
Sleep duration	-0.148	0.067	4.926	0.86 (0.75-0.99)	0.034	
Hours of sleep						0.029
< 7 h	0.469	0.177	7.038	1.60 (1.11-2.29)	0.012	
7–9 h				Ref		
≥ 9 h	0.245	0.307	0.637	1.28 (0.68-2.39)	0.431	

a Unadjusted.

b Adjusted for age, and gender.

c Adjusted for age, gender, race, BMI, education level, marital status, PIR, alcohol intake, smoking status, daily physical activity, hypertension, dyslipidemia, diabetes, CVD, and cancer or malignancy.

d Adjusted for age, gender, race, BMI, education level, marital status, PIR, alcohol intake, smoking status, daily physical activity, hypertension, dyslipidemia, diabetes, CVD, cancer or malignancy, multiple opioid use and long-term opioid use.

### Subgroup analyses

Through stratified analysis, each sample in the study was categorized and analyzed independently to ascertain the influence of confounding factors and specific population. The results suggest that certain demographic groups, including female and lower PIR, could be a significantly relevant population that is particularly vulnerable to the impact of short sleep duration, leading to an increased occurrence of depression (P for interaction < 0.05, **Table 3**).

**Table 3:** Relationship between different sleep duration and depression in subgroups of potential effect modifiers.

Subgroups	Hours of sleep			*P* for interaction
	< 7 h OR (95%CI)^#^	7–9 h OR (95%CI)^#^	≥ 9 h OR (95%CI)^#^	
**Age**				0.531
20–44 years	2.84 (1.46-5.79)*	Ref	1.56 (0.39-5.76)	
45–64 years	1.59 (1.05-2.41)*	Ref	1.22 (0.60-2.46)	
65 years older	2.35 (1.19-4.79)*	Ref	0.88 (0.22-2.84)	
**Sex**				< 0.001
Female	2.90 (1.96-4.34)*	Ref	1.05 (0.53-2.04)	
Male	0.99 (0.60-1.64)	Ref	2.18 (0.90-5.09)	
**Race**				0.361
Mexican American	3.45 (1.18-11.10)*	Ref	0.61 (0.04-5.07)	
Non-Hispanic Black	2.91 (1.34-6.68)*	Ref	0.55 (0.10-2.50)	
Non-Hispanic White	1.47 (0.98-2.21)	Ref	1.50 (0.78-2.85)	
Other Hispanic	8.76 (1.31-101.40)*	Ref	2.33 (0.06-72.90)	
Other/multiracial	6.20 (1.48-42.80)*	Ref	5.25 (0.51-56.4)	
**BMI**				0.972
Underweight	2.39 (0.99-6.03)*	Ref	0.70 (0.20-2.79)	
Normal weight	2.88 (1.34-6.43)*	Ref	0.61 (0.18-2.01)	
Overweight	1.67 (0.86-3.29)	Ref	0.86 (0.23-2.79)	
Obesity	1.76 (1.18-2.67)*	Ref	1.07 (0.52-2.17)	
**Education level**				0.210
Less than high school	1.31 (0.77-2.24)	Ref	0.51 (0.18-1.31)	
High school	2.29 (1.25-4.29)*	Ref	1.09 (0.32-3.30)	
College or above	2.29 (1.36-3.93)*	Ref	2.84 (1.17-6.78)*	
**Marital Status**				0.805
Married/Cohabiting	2.15 (1.38-3.39)*	Ref	1.38 (0.62-2.97)	
Widowed/Divorced/Separated	1.93 (1.17-3.20)*	Ref	1.25 (0.50-3.02)	
Never married	2.06 (0.72-6.35)	Ref	0.88 (0.17-4.14)	
**PIR**				0.041
PIR≤ 1.3	2.80 (1.82-4.37)*	Ref	1.43 (0.70-2.87)	
PIR (1.3 -3.5]	1.15 (0.69-1.93)	Ref	1.08 (0.39-2.80)	
PIR> 1.3	2.67 (0.94-8.29)	Ref	4.90 (0.71-29.90)	
**Multiple opioid use**				0.191
One type	1.80 (1.31-2.48)*	Ref	1.34 (0.75-2.34)	
Two types or above	3.57 (1.33-10.30)*	Ref	0.82 (0.14-4.41)	
**Long-term opioid use**				0.236
< 90 d	1.26 (0.59-2.70)	Ref	2.45 (0.51-10.10)	
≥ 90 d	2.19 (1.57-3.08)*	Ref	1.29 (0.72-2.26)	

^#^Adjusted for age, gender, race, BMI, education level, marital status, PIR, alcohol intake, smoking status, daily physical activity, hypertension, dyslipidemia, diabetes, CVD, cancer or malignancy, multiple opioid use and long-term opioid use.

*P < 0.05.

### The nonlinear analyses of the association between continuous sleep duration and depression

**Figure 2:**
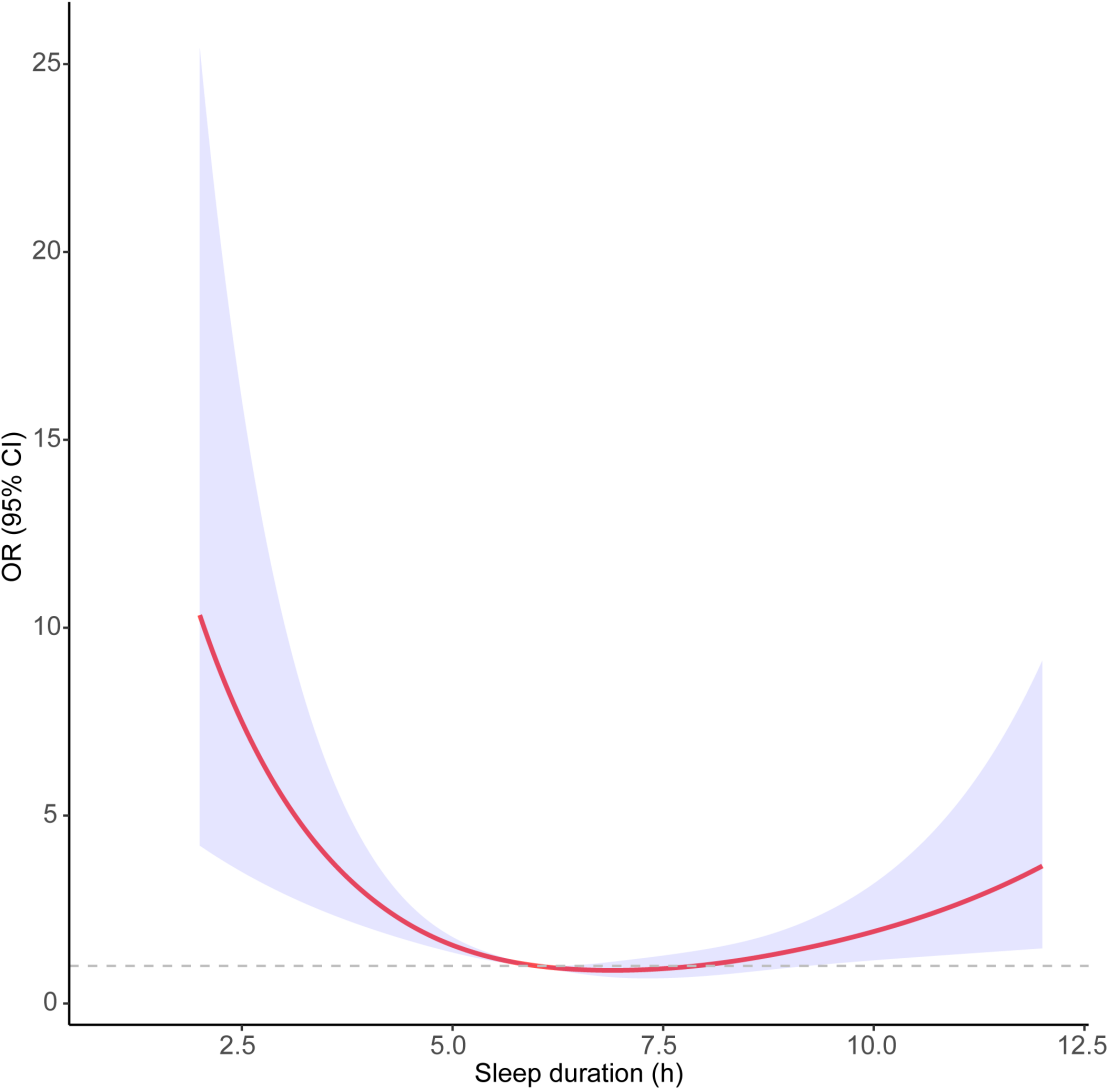
Survey-weighted restricted cubic spline analyses of the associations of sleep duration with depression.

The smoothed curve fit showed the U-shaped relationship between sleep duration and depression scores with an inflection point of 6.87 h after adjusting for all covariates (**Figure 2**). Sleeping for less than 6.87 h was connected adversely with a risk of developing depression [OR = 0.62 [95% Cl 0.46, 0.83], P < 0.001]. However, sleeping more than 6.87 h per night significantly raised the risk of depression [OR = 1.38 [95% Cl 1.06, 1.80], P = 0.011] (**Table 4**).

**Table 4:** Threshold effect analysis of sleep duration on incident depression.

Sleep duration	OR (95%CI)^#^	*P*
Inflection point	6.87	0.531
Sleep duration < 6.87 h	0.62 (0.46-0.83)	< 0.001
Sleep duration ≥ 6.87 h	1.38 (1.06-1.80)	0.011
P for log likelihood ratio		< 0.001

^#^Adjusted for age, gender, race, BMI, education level, marital status, PIR, alcohol intake, smoking status, daily physical activity, hypertension, dyslipidemia, diabetes, CVD, cancer or malignancy, multiple opioid use and long-term opioid use.

### External dataset validation

**Figure 3:**
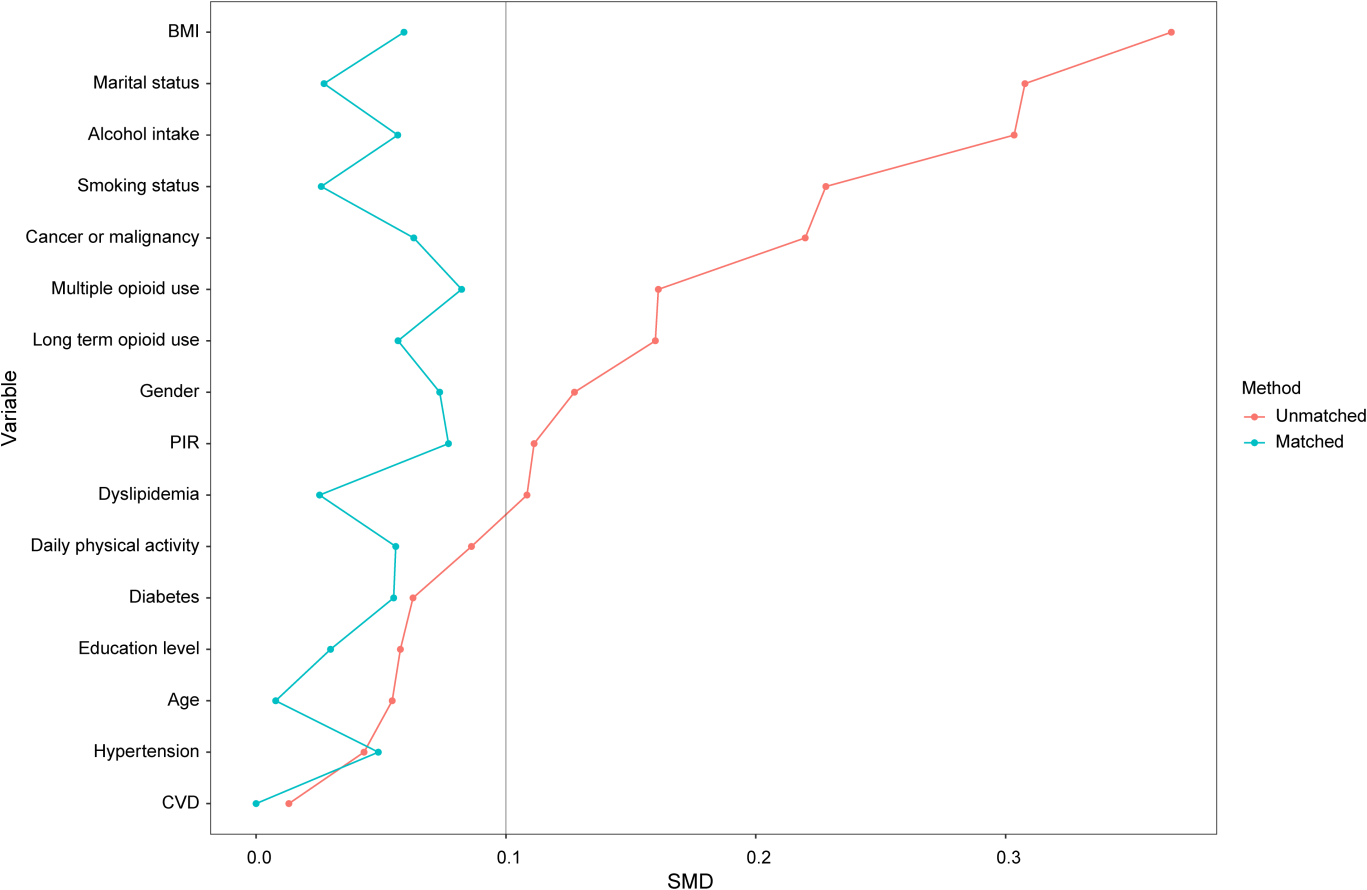
SMD between the sleep deprivation group and the non-sleep deprivation group.

In the external dataset, a total of 229 adult opioid prescription users were included, and participants were categorized into two groups based on their sleep duration: the sleep deprivation group (defined as those with sleep duration less than 6.87 hours) and the non-sleep deprivation group. The sleep deprivation group comprised 94 individuals, while the non-sleep deprivation group included 135 individuals. Following PSM, 82 participants from each group were retained in the matched cohort. Prior to matching, significant imbalances were observed across various covariates between the two groups. However, after matching, these imbalances were effectively addressed, resulting in a balanced cohort (as shown in **Supplementary Table 2**; **Figure 3**). In the matched cohort, the rate of depression was significantly higher in the sleep deprivation group compared to the non-sleep deprivation group [17 (20.7%) vs. 30 (36.6%), *P* < 0.001]. Logistic regression analysis of the matched cohort revealed that sleep deprivation was associated with a higher likelihood of depression, with a HR of 2.21 (95% CI, 1.11–4.50, *P* = 0.026). These findings suggest that sleep deprivation may be an important factor contributing to the increased risk of depression among opioid prescription users.

## Discussion

Our analysis revealed a non-linear association between sleep duration and the risk of depression among adult prescription opioid users in the study cohort. The sleep duration inflection point was identified at 6.87 hours. The findings demonstrated a U-shaped relationship, where both short and prolonged sleep durations were linked to an increased risk of depression. Specifically, in addition to shorter sleep durations, extended sleep durations also contributed to a heightened prevalence of depressive symptoms. To the best of our knowledge, this is the first study to investigate the connection between sleep duration and depression in adult prescription opioid users. Previous studies have highlighted that opioid use may adversely affect sleep quality, sleep architecture, and the ability to maintain sleep (31, 32). Additionally, individuals undergoing opioid medication therapy often report sleep disturbances, which are associated with a heightened prevalence of depressive symptoms (33). This suggests that sleep plays a crucial regulatory role in the mental health of opioid users.

Growing evidence indicates that the duration of sleep is a significant risk factor for the onset of depression. A longitudinal study (34) showed a significant association between short sleep duration and depression; while another prospective study (35) indicated no relation between short sleep and depression. The sample sizes in these studies are typically small, and the selection of covariates is often restricted. Moreover, the association between prolonged sleep and depression remains a subject of debate. While many studies investigating long sleep duration have found no significant link to the prevalence of depression (36, 37). Previous cohort studies have indicated that prolonged sleep duration is independently linked to a reduced risk of depressive symptoms (38). In contrast, a recent longitudinal study conducted in a community setting indicates that prolonged sleep duration may be linked to a higher risk of depression (39). In the initial analysis, we used 7–9 h sleep time as a normal sleep time as a reference, and found that both < 7 h sleep time and ≥ 9 h sleep time were associated with an increased risk of depression. However, when we fully adjusted for covariates, we found that only < 7 h of sleep duration was associated with an increased risk of depression. This observation may reflect underlying health conditions or lifestyle factors, rather than a direct causal relationship between longer sleep duration and depression. Consequently, after accounting for confounders, the effect of extended sleep duration appears to be reduced. Additionally, this could be due to the fact that the threshold for long sleep duration we defined was set too high, leading to a lack of statistical significance when comparing individuals with ≥ 9 hours of sleep to those with 7–9 hours. By setting the cutoff for long sleep duration at 9 hours or more, we might have included individuals whose extended sleep duration did not significantly differ from those within the 7-9-hour range in terms of depression risk. As a result, the comparison between these groups may not adequately reflect the potential impact of excessively long sleep durations beyond this threshold.

The exact mechanisms linking short sleep duration to depressive symptoms in adults using prescription opioids remain unclear, though several potential explanations have been suggested. One possibility is that insufficient sleep may worsen the sleep disturbances already induced by opioid use. Opioids are known to interfere with normal sleep patterns by decreasing the duration of deep sleep and altering REM sleep, both of which are essential for emotional regulation and cognitive functioning (40). The interplay of inadequate sleep and disturbed sleep patterns may heighten the risk of depressive symptoms by compromising the brain’s capacity to regulate emotions and stress. Additionally, reduced sleep duration may elevate stress and inflammation levels, both of which are strongly associated with the onset of depression (41, 42). Chronic lack of sleep has been associated with increased cortisol secretion and heightened inflammatory responses, which could play a role in the development or exacerbation of depressive symptoms (43). In individuals using opioids, who may already be dealing with increased stress and immune system dysfunction, the impact of insufficient sleep may be more significant. Additionally, short sleep duration frequently leads to fatigue, decreased cognitive performance, and compromised decision-making, all of which can adversely affect daily functioning and overall quality of life (44). This situation can create a vicious cycle, where diminished functioning and quality of life lead to feelings of hopelessness and despair, thus further increasing the risk of depression. For individuals using opioids, who may already face the physical and psychological challenges of addiction, the additional burden of sleep deprivation could significantly raise the likelihood of developing depressive symptoms. Furthermore, lifestyle factors linked to both short sleep duration and opioid use may also contribute to this risk. Unstable sleep patterns, poor nutrition, insufficient physical activity, and social isolation are more prevalent among those experiencing disrupted sleep and opioid dependence, all of which can exacerbate both sleep disturbances and depression. Tackling these lifestyle factors may be crucial in reducing the risk of depression in this population.

Our study also observed that the risk of depression associated with short sleep duration may be more pronounced in females and individuals from lower socioeconomic backgrounds. In women, hormonal variations during the menstrual cycle, pregnancy, and menopause can substantially influence both sleep quality and duration (45). Hormonal fluctuations can intensify the impact of insufficient sleep, increasing women’s vulnerability to mood disturbances and depression. Moreover, societal expectations and caregiving responsibilities often impose additional stress on women, further disrupting their sleep and heightening the risk of depressive symptoms when sleep is inadequate. In individuals experiencing poverty, financial insecurity, job instability, and limited healthcare access contribute to chronic stress, which is recognized to disrupt sleep patterns (46). The resulting short sleep duration can, in turn, intensify feelings of hopelessness, anxiety, and depression (47). Additionally, individuals experiencing poverty often live in environments that hinder restful sleep, such as overcrowded or noisy conditions, which may exacerbate the risk of depression linked to inadequate sleep. When sleep duration was treated as a continuous variable, the smoothed curve demonstrated a U-shaped association between sleep duration and depression scores, with an inflection point at 6.87 hours after controlling for all covariates. These results emphasize the complexity of the relationship between sleep duration and depression, underlining the importance of a detailed understanding of how varying sleep patterns influence mental health. The U-shaped curve observed in our study suggests that both short and long sleep durations are associated with a higher risk of depression, with the optimal sleep duration identified around 6.87 hours. This inflection point implies that even slight deviations from this optimal sleep duration may elevate the risk of depressive symptoms.

This study has several limitations. First, being a cross-sectional design, it cannot establish a causal link between depressive symptoms and sleep duration. Second, the sample is derived from a single country, limiting the generalizability of the findings to other regions. Third, as the data was collected through self-reported questionnaires, there is a potential for recall bias. Moreover, sleep duration in this study was assessed using a self-reported question regarding sleep on working days and reflects actual sleep time rather than subjective sleep need. However, sleep medicine distinguishes between individual sleep requirement and actual sleep duration. Some participants categorized as having “normal” sleep duration may still experience chronic sleep insufficiency if their intrinsic sleep need is higher. Furthermore, short sleep duration may partly reflect environmental or socioeconomic constraints, such as work demands or caregiving responsibilities, rather than intrinsic sleep disturbance. Because sleep was assessed only on working days, we were unable to account for discrepancies between workday and rest-day sleep (i.e., social jet lag), which may influence mood outcomes. In addition, we lacked detailed information on comorbid sleep disorders, such as insomnia or sleep-disordered breathing, which are common in individuals with chronic pain and opioid use. These factors may confound or mediate the observed association between sleep duration and depression. To further investigate the impact of sleep on depression across different domains and patterns in individuals using prescription opioids, future research should involve prospective studies with objective sleep monitoring.

Among the adults who have taken prescription opioid recently, only short sleep duration is associated with an increased risk of depression and showed a nonlinear dose-response relationship.

## Data Availability

The original contributions presented in the study are included in the article/**Supplementary Material**. Further inquiries can be directed to the corresponding author.
