# Correction: A U-shaped association between sleep duration and depression in adult prescription opioid users

**DOI:** 10.3389/fpsyt.2026.1829870

**Published:** 2026-03-23

**Authors:** Ying Li, Yuting Zhong, Wenting Guan, Luwen Liang, Yunya Zhu

**Affiliations:** 1 Department of Anesthesiology, The First Affiliated Hospital of Gannan Medical University, Ganzhou, China; 2 General Practice School of Gannan Medical University, Ganzhou, China; 3 Department of General Practice, the First Affiliated Hospital of Gannan Medical University, Ganzhou, China

**Keywords:** depression, NHANES, prescription opioid, sleep duration, statistical analysis

Author “Yunya Zhu” was erroneously assigned to affiliation 1. “Department of Anesthesiology, The First Affiliated Hospital of Gannan Medical University, Ganzhou, China”. This should have been affiliation 3. “Department of General Practice, the First Affiliated Hospital of Gannan Medical University, Ganzhou, China”.

The original version of this article has been updated.

