# Supplementary material for: A U-shaped association between sleep duration and depression in adult prescription opioid users
Source: Front Psychiatry. 2026 Mar 12;17:1755263. doi: 10.3389/fpsyt.2026.1755263 (PMC13051304; doi:10.3389/fpsyt.2026.1755263)
Supplement: Supplementary file 1 [file Table1.docx]

## **Supplementary Table 1. Drug code for prescription opioids**

| **Drug code** | **Drug name** |
| --- | --- |
| d00012 | CODEINE; |
| d00017 | MEPERIDINE; |
| d00334 | PENTAZOCINE; |
| d00360 | PROPOXYPHENE; |
| d03826 | TRAMADOL |
| d03423 | ACETAMINOPHEN; CODEINE |
| d03424 | ASPIRIN; CODEINE |
| d03425 | ACETAMINOPHEN; BUTALBITAL; CAFFEINE; CODEINE |
| d03426 | ASPIRIN; BUTALBITAL; CAFFEINE; CODEINE |
| d03430 | ASPIRIN; CAFFEINE; DIHYDROCODEINE |
| d04269 | ACETAMINOPHEN; CAFFEINE; DIHYDROCODEINE |
| d03433 | MEPERIDINE; PROMETHAZINE |
| d03434 | MEPERIDINE; PROMETHAZINE |
| d03435 | MEPERIDINE; PROMETHAZINE |
| d03676 | NALOXONE; PENTAZOCINE |
| d03682 | ACETAMINOPHEN; PENTAZOCINE |
| d04766 | ACETAMINOPHEN; TRAMADOL |
| d00308 | MORPHINE; |
| d03075 | HYDROCODONE; |
| d07453 | TAPENTADOL; |
| d00824 | OPIUM |
| d03428 | ACETAMINOPHEN; HYDROCODONE |
| d03429 | ASPIRIN; HYDROCODONE |
| d03436 | BELLADONNA; OPIUM |
| d04225 | HYDROCODONE; IBUPROFEN |
| d00233 | FENTANYL; |
| d00329 | OXYCODONE; |
| d00833 | OXYMORPHONE; |
| d00255 | HYDROMORPHONE |
| d03431 | ACETAMINOPHEN; OXYCODONE |
| d03432 | ASPIRIN; OXYCODONE |
| d00050 | METHADONE |
| d00840 | BUPRENORPHINE |
| d04819 | BUPRENORPHINE;NALOXONE |

**Supplementary Table 2. Baseline characteristics of subjects in the original and matched cohorts.**

| **Covariates** | **Before PSM** | |  | **After PSM** | |  |
| --- | --- | --- | --- | --- | --- | --- |
|  | **Non-sleep deprivation group** | **Sleep deprivation group** | **SMD** | **Non-sleep deprivation group** | **Sleep deprivation group** | **SMD** |
| N | 135 | 94 |  | 82 | 82 |  |
| **Age, years** | 66.42 (54.83-77.92) | 71.31 (62.49-80.24) | 0.370 | 72.83 (62.72-82.42) | 71.27 (62.41-80.23) | 0.076 |
| **Gender** |  |  | 0.127 |  |  | 0.073 |
| Male | 66 (48.9%) | 40 (42.6%) |  | 39 (47.6%) | 36 (43.9%) |  |
| Female | 69 (51.1%) | 54 (57.4%) |  | 43 (52.4%) | 46 (56.1%) |  |
| **BMI** |  |  | 0.366 |  |  | 0.059 |
| Underweight | 2 (1.5%) | 0 (0.0%) |  | 0 (0.0%) | 0 (0.0%) |  |
| Normal weight | 28 (20.7%) | 24 (25.5%) |  | 18 (22.0%) | 20 (24.4%) |  |
| Overweight | 44 (32.6%) | 18 (19.1%) |  | 19 (23.2%) | 18 (22.0%) |  |
| Obesity | 61 (45.2%) | 52 (55.3%) |  | 45 (54.9%) | 44 (53.7%) |  |
| **Education level** |  |  | 0.058 |  |  | 0.033 |
| Less than high school | 41 (30.4%) | 28 (29.8%) |  | 27 (32.9%) | 26 (31.7%) |  |
| High school | 41 (30.4%) | 31 (33.0%) |  | 27 (32.9%) | 27 (32.9%) |  |
| College or above | 53 (39.3%) | 35 (37.2%) |  | 28 (34.1%) | 29 (35.4%) |  |
| **Marital status** |  |  | 0.308 |  |  | 0.027 |
| Married/Cohabiting | 85 (63.0%) | 53 (56.4%) |  | 51 (62.2%) | 50 (61.0%) |  |
| Widowed/ Divorced/Separated | 32 (23.7%) | 34 (36.2%) |  | 24 (29.3%) | 25 (30.5%) |  |
| Never married | 18 (13.3%) | 7 (7.4%) |  | 7 (8.5%) | 7 (8.5%) |  |
| **PIR** |  |  | 0.111 |  |  | 0.077 |
| PIR≤ 1.3 | 62 (45.9%) | 42 (44.7%) |  | 39 (47.6%) | 37 (45.1%) |  |
| PIR (1.3 -3.5] | 44 (32.6%) | 35 (37.2%) |  | 27 (32.9%) | 30 (36.6%) |  |
| PIR> 1.3 | 29 (21.5%) | 17 (18.1%) |  | 16 (19.5%) | 15 (18.3%) |  |
| **Alcohol intake** |  |  | 0.303 |  |  | 0.057 |
| Non-drinker | 34 (25.2%) | 25 (26.6%) |  | 23 (28.0%) | 21 (25.6%) |  |
| 1-5 drinks/month | 73 (54.1%) | 58 (61.7%) |  | 48 (58.5%) | 50 (61.0%) |  |
| 5-10 drinks/month | 9 (6.7%) | 6 (6.4%) |  | 6 (7.3%) | 6 (7.3%) |  |
| 10+ drinks/month | 19 (14.1%) | 5 (5.3%) |  | 5 (6.1%) | 5 (6.1%) |  |
| **Smoking status** |  |  | 0.228 |  |  | 0.026 |
| Never | 95 (70.4%) | 56 (59.6%) |  | 51 (62.2%) | 52 (63.4%) |  |
| Current smoker | 35 (25.9%) | 33 (35.1%) |  | 28 (34.1%) | 27 (32.9%) |  |
| Former smoker | 5 (3.7%) | 5 (5.3%) |  | 3 (3.7%) | 3 (3.7%) |  |
| **Daily physical activity** | 35 (25.9%) | 28 (29.8%) | 0.086 | 20 (24.4%) | 22 (26.8%) | 0.056 |
| **Hypertension** | 79 (58.5%) | 53 (56.4%) | 0.043 | 43 (52.4%) | 45 (54.9%) | 0.049 |
| **Dyslipidemia** | 82 (60.7%) | 62 (66.0%) | 0.108 | 52 (63.4%) | 53 (64.6%) | 0.025 |
| **Diabetes** | 35 (25.9%) | 27 (28.7%) | 0.063 | 21 (25.6%) | 23 (28.0%) | 0.055 |
| **CVD** | 11 (8.1%) | 8 (8.5%) | 0.013 | 7 (8.5%) | 7 (8.5%) | < 0.001 |
| **Cancer or malignancy** | 20 (14.8%) | 22 (23.4%) | 0.22 | 16 (19.5%) | 14 (17.1%) | 0.063 |
| **Multiple opioid use** | 13 (9.6%) | 14 (14.9%) | 0.161 | 7 (8.5%) | 9 (11.0%) | 0.082 |
| **Long term opioid use** |  |  | 0.16 |  |  | 0.057 |
| < 90 d | 38 (28.1%) | 20 (21.3%) |  | 21 (25.6%) | 19 (23.2%) |  |
| ≥ 90 d | 97 (71.9%) | 74 (78.7%) |  | 61 (74.4%) | 63 (76.8%) |  |
